# The enigma of the gonadotropin-releasing hormone pulse frequency governing individual secretion of luteinizing hormone and follicle-stimulating hormone

**DOI:** 10.1016/j.xfre.2023.02.010

**Published:** 2023-04-24

**Authors:** Cornelis B. Lambalk

**Affiliations:** Department of Obstetrics and Gynaecology,Amsterdam University Medical Center, Amsterdam, Amsterdam, the Netherlands

**Keywords:** GnRH, LH, FSH, pulse frequency modulation

## Abstract

Luteinizing hormone and follicle-stimulating hormone are the two gonadotropic pituitary hormones stimulated by one hypothalamic gonadotropin-releasing hormone (GnRH) in a pulsatile way. Under several experimental conditions, it appears that a low pulse frequency promotes follicle-stimulating hormone secretion, pointing to an elegant mechanism by which, under governance of one stimulating hormone, the responses of two separate hormones can be individualized. Several experimental and fundamental studies have indicated the underlying mechanisms at the level of gene expression and post receptor events. In this article, an additional explanation is hypothetically put forward on the basis of dynamic and kinetic differences between both hormones in response to GnRH, with a key role of their difference in serum half-life combined with some GnRH-related desensitization features. Although experimentally demonstrated*,* under clinical conditions its effect remains obscure, likely because of overwhelming hormonal gonadal feedback.

With the discovery of the general principle of hypothalamic releasing hormones traveling via the portal system to induce the secretion of anterior pituitary hormones, expectations were immense with regard to identification of each of these drivers. With the known existence of two pituitary gonadotrophic hormones, luteinizing hormone (LH) and follicle-stimulating hormone (FSH), it was expected that for each, a releasing hypothalamic hormone would be discovered (1). However, the one discovered releasing hormone turned out to clearly release both LH and FSH (2). Persistent attempts to identify the separate releasing hormone for FSH were only marginally successful and unfortunately never reached clinical relevance in humans (3).

The following is a personal account of work and ideas I developed and executed some decades ago as a Ph.D. candidate while chased by my supervisors, Joop Schoemaker and Peter van Rees, to attempt to solve the intriguing issue of the separate effects of the single gonadotropin hormone releasing, which I will now refer to as GnRH, the hypothalamic secretagogue for the individual secretion of LH and FSH, and the potential key role of the frequency with which this hormone is offered to the pituitary gland. To begin, some apologies for the antique references in general and for the disproportionate, but this time unavoidable, rate of self-citations.

The key publication that ignited the awareness that there could be individual LH and FSH signaling hidden in the frequency with which GnRH was sent to the pituitary came from work from Ernest Knobil’s laboratory (4). They identified and extensively studied the crucial role of the periodicity of pulsatile secretion of GnRH. After the discovery of GnRH itself had set very high expectations for the treatment of infertility, it was disappointing and initially not understood why higher dosages both in humans and rats did not improve but rather decreased pituitary secretory gonadotropin response. However, this changed with the subsequent Eureka discovery that continuous higher dosages of GnRH caused desensitization and a key role of the pulsatile nature of its secretion, whereas until that time the observed variation of LH and FSH over the day was interpreted as a possible artifact (4, 5). The seminal experiments by Knobil (4) consisted of an ovariectomized nonhuman primate with a radio frequency destroying the hypothalamus exposed to various frequencies of exogenous GnRH with simultaneous measurement of LH and FSH in peripheral blood. Accordingly, they did not only show the dramatic declining in effect of continuous administration of GnRH for LH and FSH secretion (the desensitization effect) compared with when administered in a pulsatile fashion, but also and highly remarkably that lowering of the frequency came with a decline of LH and a “paradoxical” increase of FSH (Fig. 1), suggesting a signaling key in the frequency of GnRH stimulation for the individual regulation of the two pituitary hormones.

**Figure 1:**
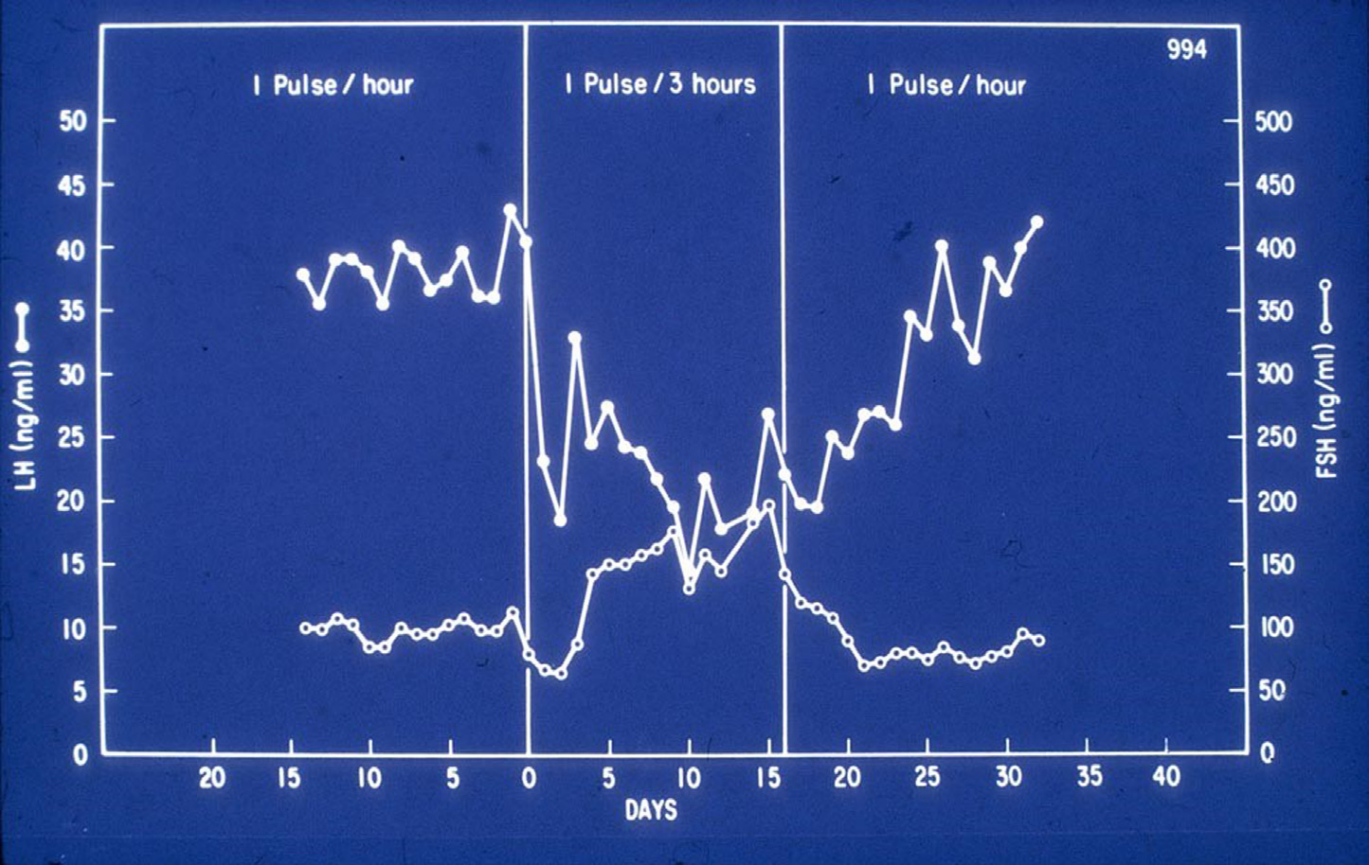
Course of luteinizing hormone (LH) and follicle-stimulating hormone (FSH) in ovariectomized Rhesus monkeys with radiofrequency lesioned hypothalamus on various frequencies of exogenous pulsatile gonadotropin-releasing hormone treatment (from (4) with permission).

This report was the start of many studies that went after the further identification of this phenomenon, the possible mechanisms, and the potential clinical implications.

There are several claims that the same phenomenon can be observed in other species. For example, in ovariectomized hypothalamo-pituitary disconnected sheep, a decrease in baseline plasma LH and mean plasma FSH concentrations increased when the GnRH pulse frequency was reduced from hourly to 2-hourly or 4-hourly (6). In addition, some studies in men with hypogonadotropic hypogonadism showed that FSH increased with the lowering of the frequency with which GnRH was substituted (7). However, other studies in men with GnRH deficiency only observed a progressive rise in LH with higher GnRH pulse frequency administration and no changes in FSH when the frequency was lowered (8).

Several fundamental studies have been undertaken to investigate potential underlying mechanisms that could explain a differential effect of the GnRH pulse frequency. The pituitary gland contains an abundance of cells that secrete both LH and FSH with their common alpha subunit and individual beta subunits. GnRH stimulates the gene expression of LH-beta, FSH-beta, and the alpha subunit.

The most widely accepted mechanism today is that varying frequencies of pulsatile GnRH stimulate distinct signaling pathways and transcriptional machinery after binding to the GnRH receptor on the cell surface of anterior pituitary gonadotropes (9), with faster frequencies favoring LH-beta transcription and slower frequencies favoring FSH-beta transcription (10). Furthermore, modulating differences in sensitivity to feedback hormones, if present, of the pituitary gonadotroph response to GnRH have been attributed. However, in the context of the latter, it should be noted that experiments and studies that did quite convincingly show the role of a lower GnRH pulse in favor of increased FSH secretion were performed under conditions without hypothalamic governance of endogenous pulsatile GnRH in combination with the absence of gonadal feedback. It should be noted that even if this subtle and intriguing control mechanism exists, there is the likely possibility that feedback mechanisms by female and male gonadal hormones may overrule the ability to observe it.

Nevertheless, the phenomenon remains a very attractive understanding for some clinical conditions that we are confronted with today, of which the reproductive endocrine profile of patients with polycystic ovarian syndrome. These patients show almost invariably elevated secretion of LH and a relative shortage of FSH. This elevated LH comes with an observed increased frequency of LH pulsatility. Nowadays, this imbalance of LH and FSH is by many assumed to be the result of an increased frequency of endogenous GnRH pulse frequency, pointing to a fundamental neuroendocrine mechanism for its origin (11).

This makes it even more relevant to question first whether the mechanism exists in humans, which has been clearly shown in several animal experimental models with artificially destructed suprapituitary structures rendering them from endogenous GnRH signaling, in combination with gonadectomy making them devoid of hormonal feedback influence.

We therefore designed an experiment many years ago for which we received formal approval from our medical ethical committee and obviously the need for written, fully informed consent to expose postmenopausally aged women with known, well defined, and identified previous suprapituitary amenorrhea to several prolonged periods of intravenous pulsatile GnRH with various frequencies. To some extent, this natural human experimental model could be considered identical to that developed by Knobil (4) in the nonhuman primate and Clarke (6) in the sheep: no hypothalamic GnRH and no gonadal feedback.

Although by design an ideal experimental model the study was hampered by many practical problems, resulting in only a soundly executed result from one patient, which was not published until now.

It involved an otherwise healthy patient born in 1954 who had deep idiopathic hypogonadotropic hypogonadism. In the past, she had undergone successful ovulation induction with intravenous pulsatile GnRH and was delivered twice. Her follow-up was characterized by persistent hypogonadotropic hypogonadism, for which she received hormonal substitution therapy until the age of 50 years. At the beginning of the experiment, she was 54 years old. Her basal serum LH and FSH levels were 1.1 and 2.6 U/L, respectively, with estradiol levels <50 pmol/L. Initially, she received 10 μg of GnRH (Lutrelef, Ferring, the Netherlands) IV via an antecubital vein every 90 minutes for 4 days using a pump (Zyklomat, Ferring, the Netherlands) to prime the pituitary gland. After that, the dose was changed to 10 μg every 180 minutes and administered for 7 days. This was followed by another 4 days of 10 μg every 90 minutes to maintain pituitary gland responsiveness, after which the 10 μg pulses of GnRH were administered every 30 minutes for another 7 days. Throughout this period, LH, FSH, and estradiol levels were measured at the beginning, at the time of each frequency switch, and when the final treatment was completed. The whole experiment lasted 26 days.

Figure 2 summarizes the essential results. LH and FSH went from hypogonadotropic to hypergonadotropic levels on the pulsatile treatment after a week with the 180-minute pulse dose, and an identical pattern could be observed after a week with the GnRH every 30 minutes with no indication that the lower frequency favored the FSH.

**Figure 2:**
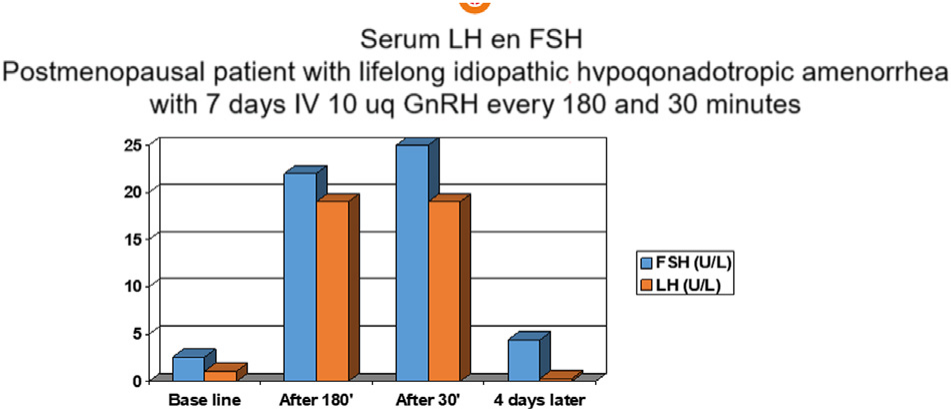
Serum luteinizing hormone (LH) and follicle-stimulating hormone (FSH) were measured before and after 7 days of intravenous pulsatile gonadotropin-releasing hormone with a pulse interval of 180 minutes and after 7 days with a frequency of every 30 minutes in a 54-year-old patient with lifelong idiopathic hypothalamic hypogonadism and postmenopausal gonadal conditions.

It has to be admitted immediately that this experiment is no proof what so ever for the absence of the phenomenon for a number of reasons. First, because of a number of setbacks, we could only study one patient. Eligible patients were rare, and there were no more patients willing to participate in this quite burdensome experiment, and moreover, the manufacturing of the required intravenous pump equipment was terminated. Therefore, we can only conclude that in this particular patient who met the ideal experimental model under the pulsatile GnRH circumstances imposed, we could not substantiate that a low GnRH pulse frequency benefited FSH secretion in an absolute sense and relative to LH.

In 1989 (12), we published a study in young women with hypothalamic amenorrhea who received three regimens of 5 days of pulsatile GnRH administered at 30-, 90-, or 180-minute intervals, with at least 6 weeks between treatments, and LH and FSH were measured every 10 minutes for 6 hours on the first and the last day (Fig. 3).

**Figure 3:**
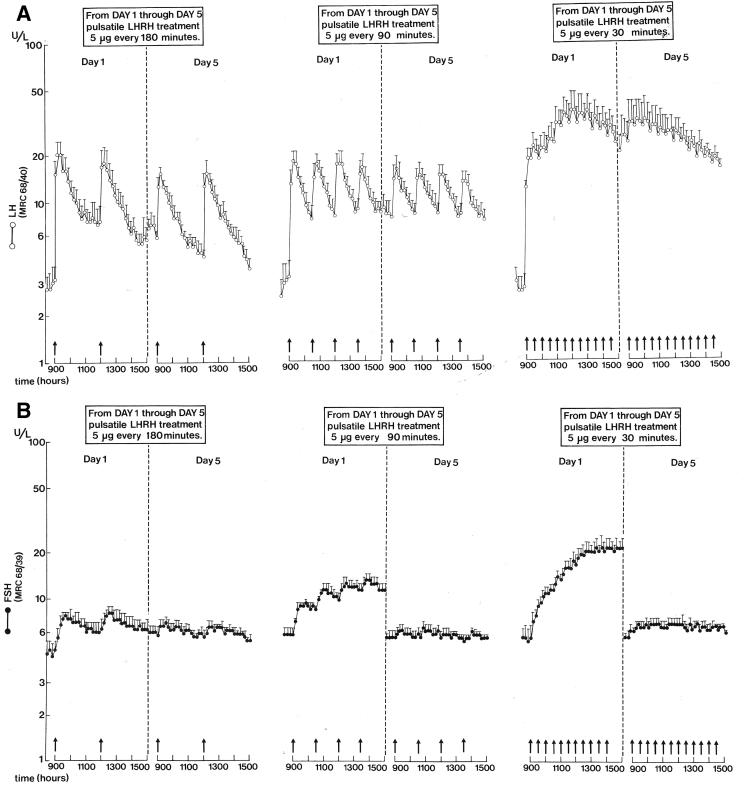
Average serum luteinizing hormone (LH) **(A)** and follicle-stimulating hormone (FSH) **(B)** were measured every 10 minutes for 6 hours on the first and the last days of 5-day intravenous administration of 5 μg gonadotropin-releasing hormone with intervals of 180, 90, and 30 minutes in young patients with hypothalamic hypogonadism (from (12) with permission).

On the first day, both LH and FSH increased in proportion to the GnRH pulse frequency. Remarkably, after 5 days, this was still the case with LH, with higher LH levels and higher pulse frequency, but not for FSH. After 5 days, FSH levels were the same at each of the administered frequencies. This study did show a clear discrepancy between the LH and FSH responses to various GnRH pulse frequencies, but not what we had expected, namely, an increase in FSH on a low-frequency GnRH pulse regimen. We concluded that under the conditions of intact gonadal feedback potential, which was in the study already overwhelmingly evident with regard to estradiol secretion after 1 day of the treatment, a possible subtle mechanism that favors FSH secretion on a low-frequency GnRH stimulus could not be observed. This could imply that its role under normal physiological conditions in the presence of active gonads may be limited. Under such conditions, a differential effect of gonadal feedback on LH and FSH in combination with GnRH pulse frequency-related changes in the pituitary’s response to it belongs to the more likely explanations.

Therefore, human studies so far do not provide hard evidence of the presence of the intriguing benefit of a low pulse frequency to FSH secretion in contrast to the mentioned preclinical animal studies. On top of that, we only have some complex fundamental biochemical work that could support its existence.

But there might be a much more obvious but hypothetical mechanism that could be an explanation.

In the past, we showed that also small single injections of GnRH cause an immediate short period of lower gonadotropin responsiveness to a subsequent GnRH injection (short-term desensitization). The phenomenon could be demonstrated in the human female and in the rat and concerns GnRH-induced LH as well as FSH secretion (13, 14). It appears that the duration of this short-term desensitization depends on the GnRH dose that caused it. The existence of this short-term desensitization or refraction after single pulses of GnRH has several consequences.

One consequence of short-term desensitization of LH and FSH might be that plasma FSH levels increase when the frequency of treatment with pulsatile GnRH is lowered. Under circumstances in which the interaction between the hypothalamus and pituitary gland is not influenced by the gonads, for example, after castration, it is hypothesized that the following may take place:

Large intervals between GnRH injections result in a large response of LH and FSH. Under such conditions, plasma levels of the hormone with the longest plasma half-life will remain higher for a longer period than those of the hormone with the shorter plasma half-life. FSH has a plasma half-life of about twice that of LH. Therefore, pulsatile GnRH treatment with a low frequency might result in higher basal FSH levels and lower basal LH levels.

This idea was tested in a graphical model and shown in Figure 4, which shows the course of two hormones with extreme differences in serum half-life (30 minutes vs. 240 minutes) over time secreted in pulses widely differing in pulse frequency (30-minute vs. 180-minute intervals). In one situation, it was assumed that each response on a pulse stimulus (for example, GnRH) was the same, and in the other situation, it was assumed that there was a temporary refraction with increased recovery after time.

**Figure 4:**
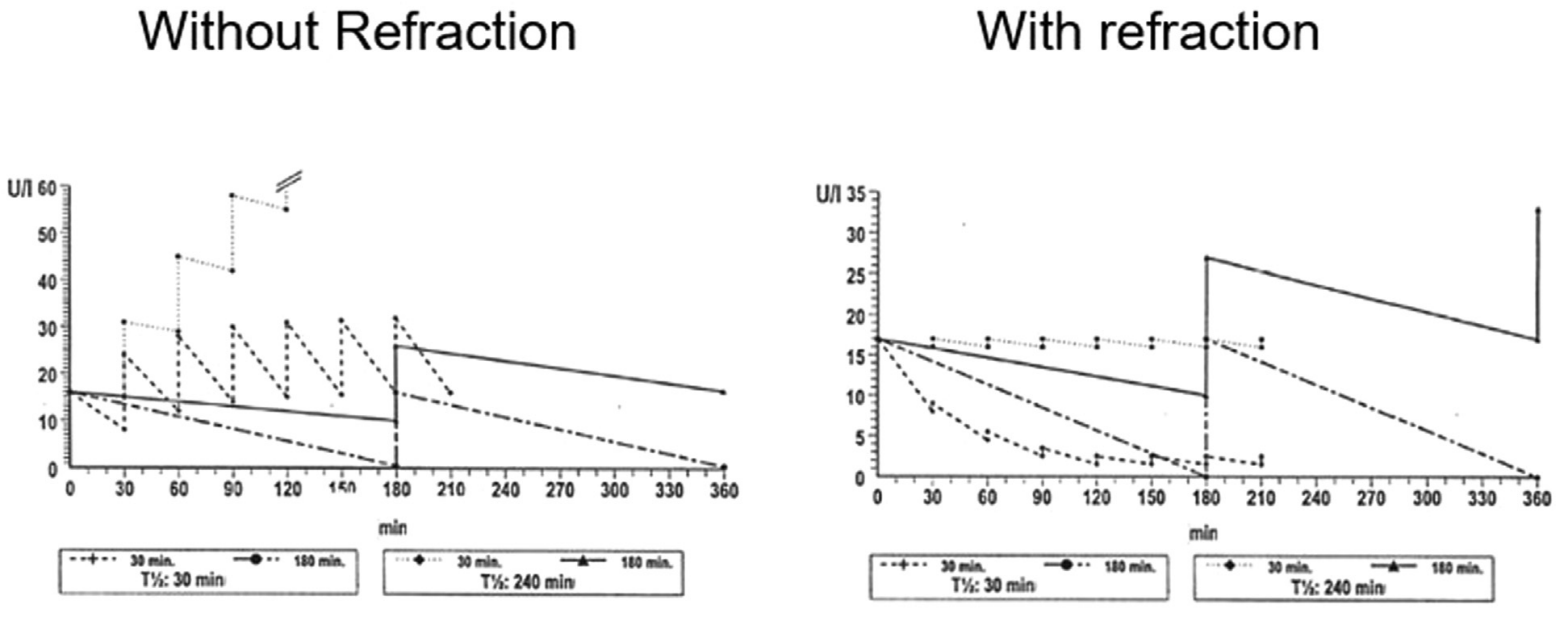
The course of two hormones with extreme differences in serum half-lives (30 minutes vs. 240 minutes) over time secreted in pulses widely differing in pulse frequency (30-minute vs.180-minute intervals). In one situation (left panel), it was assumed that each response on a pulse stimulus (for example, gonadotropin-releasing hormone) was the same, and in the other situation (right panel), it was assumed that there was a temporary refraction with increased recovery after time.

Under the conditions where temporary desensitization (refractoriness) was assumed, the low-frequency stimulus indeed increased the hormone with the long half-life and decreased the one with the short half-life. Without the assumed refractoriness in the model, this increase was in favor of the hormone with a short half-life.

For simplicity, this graphical model assumed and obviously abused some linear relationships, which are obvious oversimplifications, but it nevertheless made one point clear:

This would be the easiest and simplest mechanism of nature to elegantly direct the secretion of two hormones separately just by manipulating the frequency of the hypothalamic generator that regulates the only known GnRH of today.

Contemporary, well-equipped endocrine modeling engineers are challenged to underpin or refute the ideas suggested here.

Absence for its appearance under regular clinical conditions may have to do with FSH apparently for safety reasons being a hormone of which the concentrations need to be kept within narrow limits. Early desensitization and/or stringent gonadal feedback could account for this overruling regulation.
